# Investigation of the Effects of Plastic, Pottery, and Wooden Containers on the Microbial, Chemical, Sensory Quality, and Shelf Life of Sturgeon (*Acipenseridae*) Gut Sauce

**DOI:** 10.1002/fsn3.70748

**Published:** 2025-08-05

**Authors:** Mina Seifzadeh, Anosheh Koochakian Sabour, Ali Raoufi

**Affiliations:** ^1^ National Fish Processing Research Center, Inland Water Aquaculture Research Institute Iranian Fisheries Science Research Institute, Agricultural Research Education and Extension Organization Anzali Iran; ^2^ Student Research Committee, Department of Medicine, Faculty of Medicine, Tonekabon Branch Islamic Azad University Tonekabon Iran

**Keywords:** biodegradable containers, fermentation, fish sauce, physicochemical properties, seafood preservation

## Abstract

The sauce of sturgeon gut converts waste into value. Therefore, this study aimed to produce sauce from sturgeon gut using traditional fermentation methods and their packing in pottery, wooden, and plastic containers. The sauce packed in plastic was the control. This study assessed the effects of storage containers on the physical, chemical, microbial, sensory properties, and shelf life of sturgeon gut sauce. The fermentations were done at 30°C–35°C for 6 months. Coliform, *Escherichia coli* , molds, yeasts, and aflatoxins were not detected in both control and test samples. The highest protein (13.45%) and ash values (41.98%) were found in the pottery treatment (*p* < 0.05). Protein hydrolysis was highest in the sauce control (90%) compared to the test samples in the wooden and pottery containers (70%) (*p* < 0.05). Specific gravity (3.92) and soluble solids (34.94°Brix) values were significantly higher in sauce stored in plastic containers (*p* < 0.05). Moisture content was also highest in the plastic (47.93%) and lowest in the pottery treatments (41.29%) (*p* < 0.05). Total bacterial counts were below acceptable levels for all samples but were highest for the plastic (5.57 log CFU/g) (*p* < 0.05). Peroxide value (4.53 meq/kg oil) and Total Volatile Basic Nitrogen (94.52 mg/100 g) showed considerable change in plastic‐stored treatment compared with the other treatments (*p* < 0.05). Sensory assay and overall acceptability suggested that sauces kept in pottery containers were better. In conclusion, pottery containers present better preservation of nutritional and sensory properties and are more economical, making them an acceptable option for fermenting and storing sturgeon gut sauce.

## Graphical abstract

The study showed that fish gut sauce put in pottery jars had better nutrient value, oxidative value, and sensory acceptance, and lower microbial load compared with the other samples (wooden and plastic containers). Protein hydrolysis was highest in the sauce put in plastic containers compared to samples in wooden and pottery jars. However, the capacity for retention and shelf stability of fish gut sauce was positive, with considerations for sustainability in fermentation and storage that make pottery jars useful.

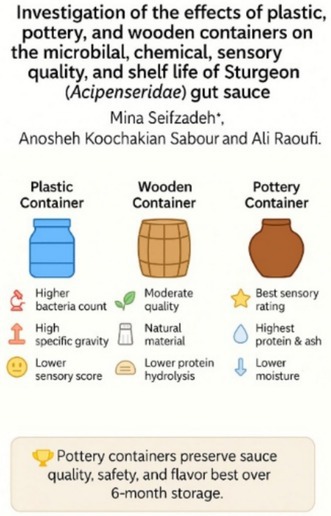

## 1 Introduction

Sturgeons are some of the most highly prized fish, while also being regarded as a major fish species, importantly placed in aquaculture and world fisheries (Xu et al. 2022). Recently, in aquaculture, the sturgeon farming industry has grown because fish species with more growth potential allow for a greater production. In Iran, sturgeon farming has expanded rapidly, beginning with, and most importantly, *Huso huso, Acipenser persicus*, and *Acipenser stellatus* , due to size/growth and acceptance of their meat and caviar in the marketplace (Seifzadeh and Raoufi 2023). Iran is ranked 23rd in the world aquaculture production (Approximately 1,352,000), which is 45% of the total fish species produced in the country (Program and Budget Office 2023). Though sturgeon fishing is only about 0.35% of the total production, its value is immeasurable in terms of the market price of caviar and sturgeon meat. However, there is a significant amount of by‐products as well because caviar extraction creates waste, mainly the viscera, accounting for an estimated 10%–15% of the fish's body weight (Seifzadeh 2024). This means that there has been an estimated output of between 377.5 to 718 tons of sturgeon waste in Iran annually, in the last couple of years (Program and Budget Office 2023).

Depending on the volume of waste produced and storage methods, fermentation can be used to store and produce products from sturgeon waste. However, fermentation is an energy‐efficient and economical method to create shelf‐stable products and culturally relevant (Yanohara et al. 2022). Further, fish sauce, in particular, has its reputation for being nutritious and having a unique flavor. Importantly, the generation of fish sauces is still largely shaped by native customs and fermentation conditions, which can contribute to extreme differences in quality, flavor, and microbial community (Seifzadeh 2024). A significant consideration, and to contribute to the fermentative processes and native customs, are the storage containers, which vary for gas exchange, moisture‐filling, microbial activity, and fermentation matrix (Ibrahim et al. 2024). Containers for storage can be plastic, pottery, or wood, and they each have their own points. Plastic containers continue to be widely used because of their cheap, convenient, and shiny surfaces; however, they create considerable health issues, environmental issues, long dedicated timeframes, possible microplastic pollution, and leaching of unwanted chemical leachate (Chaukura et al. 2021). In contrast, pottery and wood containers are both inexpensive, natural materials that are biodegradable and perhaps even advantageous to the processes involved (Bell et al. 2022; Hrcka et al. 2018).

In recent decades, studies have primarily focused on utilizing bioactive peptides and active packaging, such as edible coatings, to enhance the quality and shelf life of seafood. For example, Abdollahzadeh et al. (2023) illustrated that a nano‐chitosan coating including *Heracleum persicum* essential oil improved the microbial quality and shelf life of rainbow trout. Similarly, the use of *Anethum graveolens* seed essential oil and gallic acid in free and nano forms elevated the microbial, chemical, and sensory characteristics of minced fish during the storage period (Anvar et al. 2023). Seifzadeh (2024) applied whey protein for the preservation of frozen kilka fillets. In another research, applying whey protein with *Syzygium aromaticum* extract in nanoliposome form as an edible coating successfully forbade microbial growth and oxidative spoilage in brown rainbow trout fillets (Khosh Goftar et al. 2025). The use of dill essential oil in biopolymer‐based coatings has also proven impressive in preserving quality features of rainbow trout (Azizi et al. 2024). While these studies have seriously contributed to developing the shelf life of seafood, most have noticed the efficacy of additives and coatings rather than the direct and comparative impact of various storage containers. It also appears that edible coatings do not apply to the processing and shelf life of aquatic waste.

An important gap in the literature is the absence of comprehensive and comparative studies on the effects of general storage containers, like plastic, clay, and wood, on the microbial, chemical, and sensory characteristics, as well as the shelf life of seafood by‐products and their products, such as sturgeon gut sauce. Simultaneously examining these aspects could play a major role in optimizing packaging and storage procedures, ultimately resulting in higher‐quality, longer‐shelf‐life products. The innovation of this study lies in its simultaneous investigation of three commonly used container types on diverse properties in the sauce product. The outcomes of this study could present new insights for choosing optimal packaging materials in the seafood processing industry and serve as a scientific basis for expanding value‐added products from aquatic by‐products. We hypothesize that the container type considerably influences the maintenance and quality of sturgeon gut sauce, with clay and wooden containers potentially presenting a better condition compared to plastic. Therefore, the available study objectives are to conduct a comprehensive comparison of three storage container types, including plastic, clay, and wood, on the microbial, physicochemical, and sensory features and shelf life of sturgeon gut sauce over the storage period.

## 2 Materials and Methods

### 2.1 Reagents and Materials

Whole of chemicals and reagents applied in this study were of analytical grade. Salt (Iran) was food food‐grade and filtered white granule. Filtration soil or Tonsil (Activated bleaching clay) was obtained from Sigma‐Aldrich (Germany). Whatman No. 1 paper (Cytiva, UK) was utilized for filtration. The solvents and reagents used for chemical and physical experiments were from Merck (Germany). Microbial culture media for microbiological assays were from Quelab (USA).

### 2.2 Sampling

Intestines from cultured Sturgeon, including *H. huso, A. persicus*, and *A. stellatus* (For the treatment, three individuals per species, each approximately 7 kg in weight) were obtained from a Sturgeon processing factory. Under washing and cutting, the intestines were separated and put in Styrofoam chests containing crushed ice (At a ratio of 2:1 ice to waste weight). Samples were transported to the Processing Dep of the International Sturgeon Research Institute (Rasht, Iran) for processing. The intestines were washed using chlorinated water (Dissolving 100 ppm sodium hypochlorite in water) with a contact time of 10 min, then washed with drinkable water. Later, they were split into small‐scale, uniform pieces.

### 2.3 Processing

A total of 6 kg of gut waste (2 kg from each species) was used for the sauce preparation. The treatments included:

Pottery container: 6 kg of intestine + 25% pure salt + an unglazed pottery container. These materials are made from alluvial clay that is rich in calcium and iron, sourced locally and fired at 950°C. The earthenware properties must comply with EU Directives 1935 (2004) and 2023 (2006), which specify that earthenware must be inert, food‐grade, and free from heavy metals such as lead or cadmium.

Wooden container: 6 kg of intestine + 25% pure salt + wooden container. These were crafted from a hardwood, oak ( *Quercus robur* ). In terms of hygiene, the wood characteristics followed the ISPM 15 (International Standards for Phytosanitary Measures) (FAO 2002).

Plastic container: 6 kg of intestine + 25% pure salt + plastic container. They were made from food‐grade polyethylene with a thickness of approximately 0.5 mm (This group was the control).

Each container has a nearly 8 L capacity. They were cylindrical, approximately 25 cm in height × 20 cm in diameter. Containers were cleaned in 2 stages, including washing with detergent, rinsing, sterilization with 70% ethanol, and air‐drying. The 25% salt was added to the intestines (W/W). After mixing, they were packed into related containers. Containers were saved at 30°C–35°C in a ventilated region under ongoing white fluorescent lighting (Free of sunlight) for 6 months. The initial pH of the intestine was 7 (Koochakian Sabour 2000). After the fermentation period, the remained material was removed from the sauce using a cylindrical metal mesh (2 mm pore size). The hydrolysate (Liquid phase) was filtered via muslin lace, followed by bulky cotton fabric and tonsil clay filtration (Filtration soil) to eliminate pigments and small particulates. In the ultimate phase, Whatman filter paper was applied for tiny particles. The filtered sauce was transferred into clean pottery, plastic, and wood containers. For pasteurization, containers containing the sauce were submerged in an 80°C water bath for 30 min, followed by quick cooling to room temperature. Sauce samples were packaged in quantities of 500 g per container.

### 2.4 Storage and Sampling

The pasteurized sauce treatments were preserved in a refrigerator (4°C ± 1°C) for 6 months. Nutritional, microbial, chemical, physical, and sensory analyses were performed monthly. Each treatment was examined in triplicate per sampling time, resulting in 21 samples per group over 6 months and zero time.

### 2.5 Experimental Design

A completely randomized design (CRD) with one‐way ANOVA statistical analysis was used. The design had three kinds of containers (Pottery, plastic, and wooden) as treatment categories. Each trial was conducted three times per sampling time (7 times), and samples were assayed monthly to measure treatment‐based results.

### 2.6 Nutritional Composition

Protein value was measured using the macro‐Kjeldahl (Behr‐Gerhardt, Germany) with a conversion factor *N* × 6.25. For digestion, 8 g of catalyst and 25 mL of concentrated sulfuric acid were added to the digestion flask of the apparatus, including 2 g of the sample. The acquired transparent, greenish solution was distilled with distilled water (two‐thirds of the flask volume) and a few boiling stones. The ammonia vapors were accumulated in an Erlenmeyer flask, including 50 mL of 2% boric acid and 3–4 droplets of bromocresol green indicator. It was titrated with 0.1 N sulfuric acid (AOAC 2005).

Fat value was measured using the acid hydrolysis procedure and Soxhlet extractor (5AK‐Behr, Germany). 50 mL of 4 N hydrochloric acid was mixed with 5 g of the sample. The blend was warmed in a water bath at 80°C for 1 h, then filtered via Whatman filter paper. The filter paper was moved to a Soxhlet extractor. A weighed flask, containing hexane (Two‐thirds of its volume), was attached, and extraction was performed for 6 to 8 h (AOAC 2005).

Ash amount was determined using the gravimetric procedure. The primary weight of clean crucibles was obtained after heating in a muffle furnace (Tech Fine, Korea) at 600°C for 1 h. 10 g of the undried sample was moved to the crucible, and the samples were heated at 550°C for 12 to 18 h. After cooling and weighing of the crucibles, the ash content was calculated (AOAC 2005).

Moisture amount was determined using the drying procedure in an electrical oven (Memmert, Germany). Ten grams of sauce were put in a pre‐weighed Petri dish and dried at 100°C until reaching a constant weight. The Petri dish was cooled in a desiccator and reweighed. The moisture amount was accounted for according to the weight loss during drying (AOAC 2005). All devices used in nutritional value determination were calibrated.

### 2.7 Microbial Analyses

Standardized approaches were used to evaluate the microbial quality of fish sauce samples to identify the effect of each treatment on its microbial safety and stability. The total viable bacterial count was measured using the pour plate method on Plate Count Agar (Maturin and Peeler 2001; Andrews and Hammack 2003). While *E. coli* (Anaerobic facultative, surface method) and total coliform (Anaerobic facultative, pour plate method) counts as indicators of fecal contamination were assessed on selective media MacConkey agar and Eosin Methylene Blue Agar, respectively (Feng et al. 2002). Anaerobic bacteria were measured in anaerobic conditions using culture medium (Sulfite Polymyxin Sulfadiazine Agar, pour plate), as described by Solomon and Timothy Lilly (2001). Fungal (mold and yeasts) counts (Aerobic, surface method) were made on Sabouraud Dextrose Agar, incubated at 25°C for 5–7 days, to determine fungal contamination and potential for spoilage, as described by Tournas et al. (2001). Detection and enumeration of Staphylococcus species (Anaerobic facultative, surface method) used Baird‐Parker agar supplemented with egg yolk tellurite, based on the method described by Bennett and Lancette (2001). Serial dilutions were plated (10^−1^ to 10^−3^). Total bacterial counts, coliform, E. coli, and Staphylococcus bacteria cultures were incubated at 37°C for 48 h. Results are shown as the logarithm of colony‐forming units per gram (log CFU/g) of sample. Standard laboratory QA/QC procedures were followed from the beginning of all cultures, including triplicate plating. Quality assurance procedures were implemented during the entire study, including positive control, negative control, and standardized inoculation procedures, to record valid and reproducible results. Standard detection limits for standard microbiological methods include < 30 CFU/g analyte for a 25 g food sample. Low levels of contamination are less than 10 CFU/g. All devices used in the microbial analysis were properly calibrated.

### 2.8 Physical Analyses

Quantitatively, the physical properties of the sauce were measured using standardized equipment and methods.

The specific gravity was determined volumetrically by weighing the sauce with a Metter AE200 calibrated (Metter Toledo, USA). It measures density with respect to water.

The pH was measured using a digital pH meter. A 10‐fold dilution was prepared using 5 g of sample with distilled water and allowed to sit at room temperature for 30 min before measuring pH (FAO 1986). The pH meter was calibrated.

The color was assessed using the Hunter Lab Colorflex system (USA), which determines the color parameters in Lab* space with L* indicating lightness; a* indicating the red‐green axis; and b* indicating the yellow‐blue axis. The Hunter Lab used was calibrated.

The degree of hydrolysis (DH) of protein was understood as the amount of nitrogen soluble in trichloroacetic acid (TCA); therefore, since TCA precipitates protein, the soluble fraction contains smaller peptides and amino acids, whose nitrogen was determined. One part of 10% TCA was added to 1 part of the sauce. The TCA and sauce mixture was centrifuged at 10,000 rpm for 10 min at 4°C. The nitrogen content of the supernatant was measured by Kjeldahl (AOAC 2000). This property was measured once at the end of the fermentation period to measure the amount and efficiency. All devices used in protein hydrolysis analysis were calibrated.

Soluble solids (°Brix) were assessed with a refractometer (Carl Zeiss IMT Corp, USA) by depositing a few drops of the sample onto the refractometer slide; each treatment group was measured (Mancini et al. 2023). Salt absorption, specific gravity, and°Brix were also evaluated metrics at the start and end of storage to allow comparisons in the temporal changes of these physical properties. All devices employed to conduct the physical measures were calibrated.

### 2.9 Chemical Examination

The salt amount was measured using a salinometer calibrated with a potassium chloride solution of known concentration. All devices used in salt analysis were calibrated.

The peroxide value (PV) was determined using the iodometric titration procedure. A 3 g specimen was blended with 30 mL of an acetic acid–chloroform solution (3:2, v/v) and warmed at 60°C for 3 min. The mixture was filtered via Whatman No. 1 paper. 1 mL of potassium iodide solution was mixed with the filtrate, and it was shaken strongly. Titration was performed with 0.01 N sodium thiosulfate using 1% starch as an indicator, and the endpoint of the experiment was when the blue color vanished (FAO 1986). All devices used in PV value analysis were calibrated.

The Thiobarbituric acid reactive substances (TBARS) value was determined using a colorimetric procedure. A total of 50 mL of distilled water was mixed with 10 g of the specimen. At the next stage, 47.5 mL of distilled water and 2.5 mL of 4 N HCl were added until the pH reached 1.5. Following distillation (10 min), the distillate was mixed with a TBA indicator and heated for 35 min. The obtained mixture was centrifuged, and the absorbance of the supernatant was read at 532 nm (FAO 1986). All devices used in TBARS analysis were calibrated.

Total volatile base nitrogen (TVB‐N) was examined using the Kjeldahl method. Ten grams of the specimen were mixed with 2 g of magnesium oxide, 300 mL of distilled water, and a few glass balls. The receiving flask contained 25 mL of 2% boric acid and a few droplets of 0.1% methyl red. The digestion flask was heated (10 min) until boiling began and continued for 25 min of distillation. The distillate was titrated with 0.1 N sulfuric acid until a red color was reached (FAO 1986). All devices used in TVB‐N analysis were calibrated.

Along the lines of Stroka et al. (2000), high‐performance liquid chromatography (HPLC) was used to quantify aflatoxin in fish sauce. At room temperature, fish sauce was left undisturbed for 30 min. At the next step, the 5 g sample was integrated with 1 g of salt and 25 mL of extraction solvent, including methanol and distilled water, 55:45 v/v. It was centrifuged for 10 min at 4000 rpm and then 10 min at 7000 rpm. The supernatant, obtained by filtration through Whatman No. 1 paper, was applied to the experiment. A filtrate of 15 mL was added to 30 mL of distilled water and was filtered once more. Thirty milliliters of the filtrate were run on the aflatoxin‐specific immune affinity column at a flow rate of 2–3 mL/min. The column was rinsed with 10 mL of distilled water and dried by positive air pressure. After this step, 500 μL and 1000 μL of methanol were eluted from the column. The elute was diluted with 1500 μL of distilled water and blended totally. A 100 μL aliquot of the solution was injected into the HPLC (Detection limits: AFB1 and AFG1 = 1 μg/kg; AFB2 and AFG2 = 0.5 μg/kg). All devices used in aflatoxin analysis were calibrated.

### 2.10 Sensory Evaluation

For the sensory assay, 10 g of fish sauce was placed into 20 mL glass vials with screw caps and placed at ambient temperature for 30 min. Specimens were selected at random and coded with three‐digit numbers. The trained panel, consisting of 30 males and females between the ages of 30 and 40, performed monthly sensory analyses on a 5‐point hedonic scale. The panelists were selected from the Department of Food Science. They were screened for sensory aptitude and trained for 3 h to recognize fish sauce attributes. Thus, five attributes were evaluated, including color, odor, appearance, presence of foreign particles, taste, and overall acceptability. These properties were rated 5 (Excellent), 4 (Good), 3 (Acceptable), 2 (Poor), and 1 (Unacceptable) (Gilbert 2013; Codex Alimentarius Commission 2018).

The appearance of the sauce shows its optical features that can be recognized without tasting or smelling. This includes different agents like color consistency (a uniform color, discoloration, streaks, and uneven tones), clearness or dimness, and whether it has a favorable opalescence or seems dark or separated.

Alien particles are mentioned as obvious such as insect fragments (Codex Alimentarious Commission 1995), non‐hydrolyzed remainders or insoluble solids. The presence of foreign or undesirable particles was determined by visual inspection, spectrophotometric analyses at specific wavelengths [600 nm], and light microscopic examination for impurities, bone fragments, fibers, undigested pieces, or mold.

Assessment meetings lasted 30–45 min and were conducted monthly. All devices used in sensory analysis were calibrated.

Environmental situations in the sensory laboratory were standardized by temperature: 22°C ± 1°C, moisture: 50%–60%, lighting: white fluorescent light (500 lx), and room girths: 4 × 5 m. Sensory assays were performed in an isolated, sound‐free, and odor‐free surrounding. Analysis was conducted in separate small rooms.

### 2.11 Statistical Analysis

The results from bacterial, chemical, physical, and sensory analyses of sauce treatments were checked using SPSS version 25. A one‐way ANOVA was performed to make comparisons of the averages across the sauce treatments. A two‐way ANOVA was studied to examine the interaction of pottery, plastic, and wooden packaging (Types of packaging) and storage time. If the obtained results of the one‐way and two‐way ANOVA were considerable, Duncan's multiple range test statistical method was applied for post hoc differences. The Shapiro–Wilk and Levene's tests determined the normality of the results and the equality of variances, respectively. The remarkable level was at *p* < 0.05. Sensory outcomes were considered as interval results and were checked using non‐parametric Kruskal‐Wallis.

## 3 Results and Discussion

### 3.1 Nutritional Composition

As exhibited in Table 1, the mineral amount (Ash) was highest in the pottery container (41.98%) and lowest in the plastic container (38.37%) (*p* < 0.05). It did not change in the wooden and plastic containers (39.69% and 38.37%) (*p* > 0.05). It did not have significant differences among the plastic (38.11%–38.37%), wooden (39.54%–39.69%), and pottery (41.63%–41.98%) treatments during the storage period (*p* > 0.05), because mineral concentrations are relatively unaffected by storage conditions. The higher ash content in earthenware treatments may be attributed to mineral leaching from the porous walls of the container into the fermenting product. Previous studies have shown that minerals such as calcium, magnesium, and trace elements can migrate from ceramic materials into food under acidic conditions (41.98%). In addition, high hydrolysis of protein causes the expansion of dissolved solids in the liquid phase. These are details that may influence the value of ash (Koochakian Sabour 2000). Yanohara et al. (2022) generated the sauce from the ground inner parts of white sturgeon by innate proteases at 50°C within 24 h. The ash quantity was 1.01% in the autolysis liquid. The results of this study are not consistent with our study. They relate to the hydrolysis degree, type of primary material, salt concentration, fermentation time, genus of container, and the presence of other ingredients in the processing.

**TABLE 1:** Nutritional value of sturgeon intestine traditional sauce stored in plastic, pottery, and wooden containers.

Index treatment	Protein (%)		Fat (%)		Ash (%)		Moisture (%)	
	Zero time	Sixth month	Zero time	Sixth month	Zero time	Sixth month	Zero time	Sixth month
Wooden	9.85 ± 1.39^aB^	10.28 ± 1.47^aB^	4.12 ± 1.13^aA^	4.29 ± 0.94^aA^	39.54 ± 1.15^aB^	39.69 ± 1.44^aB^	46.49 ± 1.45^aB^	45.74 ± 2.13^bB^
Pottery	13.11 ± 1.56^aA^	13.45 ± 1.87^aA^	3.02 ± 1.17^aA^	3.28 ± 0.78^aA^	41.63 ± 1.24^aA^	41.98 ± 1.85^aA^	42.24 ± 1.36^aC^	41.29 ± 1.59^bC^
Plastic	9.39 ± 1.63^aB^	9.51 ± 1.40^aB^	4.15 ± 1.16^aA^	4.27 ± 0.91^aA^	38.11 ± 1.27^aB^	38.37 ± 1.15^aB^	48.35 ± 1.24^aA^	47.93 ± 1.97^aA^

*Note:* The presented letters in the same column present no significant difference (*p* > 0.05). Lowercase letters indicate differences between treatments. Uppercase letters indicate statistical differences for each treatment separately during storage time (Container effect).

Protein content was greatest in the pottery container (13.45%) and smallest in the plastic container (9.51%) (*p* < 0.05). In all cases, the initial protein content decreased over time, shown in sauce stored in pottery (13.11%–13.45%), wooden (9.85%–10.28%), and plastic (9.39%–9.51%) containers. This can be associated with microbial action (*p* > 0.05). Based on Table 1 the sauce contained high protein content (9.51%–13.45%). It can be due to the autolysis of the sample during fermentation. Greatest protein extraction happens in the pH 7–9 limit, which is not in the test treatments. However, the accumulation of bacteria was higher in the plastic treatment than in other treatments. Therefore, considering the bacterial role in fermentation, this phenomenon is justifiable (Seifzadeh et al. 2025). So, earthenware vessels seem to preserve the nutrient components of sturgeon gut sauce better than wood or plastic containers. The wooden vessels had some nutrient reduction, and the lowest nutrients were in the plastic treatment. Generally, this could explain that plastic possibly presents greater potential for microbial activity or less favorable environmental conditions for more protein degradation or leaching. Additionally, plastic containers have no buffering capacity and are devoid of mineral content. This could account for less‐than‐optimal conditions to support protein stabilization. Clay and wood, as primary materials, can create a physical barrier to the prevention of microbial spoilage and could support proteolytic enzyme activity with more favorable environmental conditions (Such as porosity and oxygen diffusion properties of containers). Puat et al. (2015) accounted for the protein value in fish sauces (Kecap Ikan and Nampla) as 1.60% and 10.85%, respectively. Peñafiel and Escriba (2023) displayed that the protein content of fish sauce was 1%–2.96%. Yanohara et al. (2022) generated the sauce from the ground inner parts of white sturgeon by innate proteases at 50°C within 24 h. The protein was 14.10% in the autolysis liquid. The various constituents of protein in the fish, as the raw substance, generate diverse amounts of protein in the sauce. Also, the unlike protein value in the sauce may be due to the loss of soluble protein during washing with water and the dilution of the cached liquid from fish fermentation (Peñafiel and Escriba 2023).

Fat presented the highest content in the wooden container (4.29%) and the lowest in the pottery (3.28%). Fat value between treatments (3.28%–4.29%) showed no remarkable difference (*p* > 0.05). Fat exhibited a slight decrease in pottery (3.02%–3.28%), wooden (4.12%–4.29%), and plastic (4.15%–4.27%) containers (*p* > 0.05), possibly related to oxidation or the action of lipases from texture and live and dead bacteria. The fat in the experimental samples was approximately low (3.28%–4.29%), which can be impacted by the salt. In addition, dissimilar acids were produced during the hydrolysis of the sauce. As fat has an alkaline property, it is neutralized by these acids. Also, the reduction in fat content in earthenware containers (3.28%) may be partially explained by the natural porosity and adsorptive properties of clay, which can bind lipid molecules (Irto et al. 2022). The fat quantity was 2.83% in the obtained autolysis liquid from the ground inner parts of white sturgeon by innate proteases at 50°C within 24 h by Yanohara et al. (2022). The results of this study are not consistent with our study. This difference can be related to the genus of the container, the various contents of generated acids, and different amounts of salt.

Moisture ranged from 41.29% (pottery) to 47.93% (plastic) (*p* < 0.05). Moisture showed slight fluctuations in pottery (41.29%–42.27%) (*p* < 0.05), wooden (45.74%–46.94%) (*p* < 0.05), and plastic (47.93%–48.35%) jars (*p* > 0.05). In contrast to plastic containers, clay and wooden containers are rather likely to cause moisture damage, related to humidity in the refrigerator, the thickness of the plastic, the moisture maintenance ability, vaporization, absorption of moisture into the wooden container, and the porous properties of pottery, to absorb or evade moisture. The moisture reduction may be due to the use of salt in the sauce production (Nakano et al. 2018). Yanohara et al. (2022) generated the sauce from the ground inner parts of white sturgeon by innate proteases at 50°C within 24 h. The protein, fat, moisture, and ash quantities were 14.10%, 2.83%, 80.16%, and 1.01% in the autolysis period. Nakano et al. (2018) explained that the humidity was 49.65%–62.15% in the salmon sauce produced from flesh, viscera, an inedible section, and soft roe. Our moisture outcome was opposite to the recent study. It may be due to dissimilar salt contents (10% and 25%), fermentation period, and processing procedures (Peñafiel and Escriba 2023).

Preservation of food value is one of the major advantages of earthenware. Due to the pores in earthenware that regulate heat and humidity during storage, nutrient retention appeared higher in earthenware containers, possibly due to reduced leaching and stable pH conditions.

### 3.2 Microbiological Quality

Based on Table 2, total bacterial counts increased during storage time in sauce treatments but were below the allowable range (7 log CFU/g) determined by the Center For Food Safety (2014). Sauce stored in plastic vessels showed the greatest total bacterial counts (5.57 log CFU/g), compared to samples stored in wooden and pottery containers (2.51 and 2.28 log CFU/g, respectively) at the termination period (*p* < 0.05). *E. coli* , Coliform, Staphylococcus, anaerobic bacteria, and aflatoxin were not detected in any sauce treatments at the end of the storage period. Total bacterial counts presented remarkable differences during storage in the sauce stored in pottery (1.75–2.28 log CFU/g), wooden (1.69–2.51 log CFU/g), and plastic (4.19–5.57 log CFU/g) containers (*p* < 0.05). Variation in microflora between container types may also depend on the physicochemical properties of the bulk materials of those containers. For instance, earthenware is porous, providing a secondary means for low‐level oxygen diffusion (micro‐oxygenation), potentially favoring aerobic or microaerophilic microorganisms during the fermentative process. Microbial products defined as metabolites with antimicrobial properties may also highly influence microbial succession, due to plain coincidence in their abilities to adsorb into the porous matrix, while further altering inhibitory concentrations at a local level. The solid structure of clay, to either generate oxygen or release finely ground minerals (e.g., aluminosilicates and nitrous compounds), either in the material of the container or with the naturally sourced clay, may influence microbial composition as well. On the other hand, the topographical trait and the orientation of a plastic container's surface (Finished flat or with minor undulations) may offer an example of shape accumulation or biofilm accumulation, which may further favor bacterial accumulation. The compounds used to process the sauce have several roles in the microbial safety of the sauce. For example, salt diminishes water activity and barricades the activity of spoilage microorganisms by depriving water via osmosis (Lestari et al. 2023). However, it provided the situations for the function of salt‐tolerant bacteria. Staphylococcus bacteria were not looked at in the treatments. The Staphylococcus bacterium can remain in 20% salt. The salt quantity was not acceptable in the experimental sauces for their growth at fermentation termination. However, Staphylococcus bacteria are the inherent flora of the skin of humans, but since gloves were applied throughout processing, this bacterium did not enter the sauce. Coliform and *E. coli* can flourish at 8% salt (Vos et al. 2011). However, these bacteria were not seen in the available study, which is related to the usage of chlorinated water and gloves throughout production. The open pores of earthenware allow for the evaporation process, so the product stored in the earthenware will cool naturally. Also, wooden containers are able to keep the product cold. Decreasing the temperature of the product changes the development and activity of bacteria and occasions their actuality to reduce. Therefore, the bacterial number in ceramic and wooden containers diminished.

**TABLE 2:** Microbial indexes of sturgeon intestine traditional sauce are stored in plastic, pottery, and wooden containers (log CFU/g).

Index	Total bacterial counts		
Treatments sampling time (month)	Pottery	Wooden	Plastic
Zero	1.75 ± 0.25^bB^	1.69 ± 0.48^bB^	4.19 ± 0.49^eA^
1	1.78 ± 0.27^bB^	1.88 ± 0.37^bB^	4.25 ± 0.38^deA^
2	1.87 ± 0.36^abB^	1.97 ± 0.29^bB^	4.41 ± 0.79^dA^
3	1.91 ± 0.55^aB^	2.16 ± 0.43^abB^	4.68 ± 0.68^cdA^
4	2.01 ± 0.42^aB^	2.25 ± 0.59^aB^	4.94 ± 0.63^bcA^
5	2.14 ± 0.39^aB^	2.37 ± 0.77^aB^	5.21 ± 0.74^abA^
6	2.28 ± 0.45^aB^	2.51 ± 0.64^aB^	5.57 ± 0.76^aA^

*Note:* The same letters in the same column and row show no significant difference (*p* > 0.05). Lowercase letters indicate differences between treatments. Uppercase letters indicate statistical differences for each treatment separately during storage time (Container effect).

Plastic containers, on the other hand, are a suitable substrate for bacterial aggregation. The surface properties of plastics show smooth surfaces (Easier for adhesion) and hydrophobicity (To form biofilms). Furthermore, microbes and bacteria tend to be placed on surfaces that have greater density and are non‐porous (Plastic), rather than those that are more porous. Also, stability and resistance to degradation cause the microbial community to remain. Also, they do not cool the product, which causes the bacteria to continue their activity and increase in number. Earthenware is made of clay. Clay inhibits the growth of certain types of bacteria (Koochakian Sabour 2000), acting as a barrier against bacteria that prevents spoilage. Reducing temperature creates favorable conditions for the growth and activity of psychrophilic bacteria and fungi, which were not observed in the present study. The salt value in the investigational treatments was not appropriate for the mold and yeast function. According to our outcomes, anaerobic bacteria were not identified in the sauce groups. Salt removes oxygen from the tissue of the samples and creates the conditions for the activity of anaerobic bacteria. On the other hand, the used fish were cultured in cement pools and did not feed on benthic creatures, so they were not contaminated with anaerobic bacteria (Seifzadeh et al. 2025). Sakpetch et al. (2022) explained that proteolytic (From 3.51 log CFU/g to 5.34 log CFU/g) and lipolytic (From 2.98 log CFU/g to 4.41 log CFU/g) bacteria were increased until 60 days of fermentation. Also, in the available study, the total bacterial counts increased and achieved 2.28–5.557 log CFU/g at the end of the storage period. It is principally related to the nutrients, peptides, and free fatty acids production, the hydrolysis degree, and the environment being satisfactory for halophilic bacteria growth and their function (Hu et al. 2020).

### 3.3 Physical Properties

As detailed in Table 3, the specific gravity was a higher level for the sauce stored in a plastic container (3.92) than in the pottery (2.68) and wooden treatments (2.52) (*p* < 0.05). This indicator was almost stable during the keeping cycle. It may be due to the steady storage situation, packaging, and intensity of heat. The specific gravity can change for multiple symptoms. Usually, the specific gravity varied with the balance of lipid and air. It may be affected by the temperature of the components, the environment, and the packaging. The sauce samples were not the same given temperature and packaging positions (Koochakian Sabour 2000). Hjalmarsson et al. (2007) defined the comparative specific gravity of summer sauce capelin to be 1.14 after fermentation of 270 days. Specific gravity was within a narrow limit (2.52–3.92) among plastic, pottery, and wooden treatments during storage periods (*p* < 0.05). Our outcomes contrast with Hjalmarsson et al. (2007), which reported greater contents of soluble solids. These differences in results indicate that not only soluble solids, but also different types of other dissolved solids (Like protein degradation products), affected specific gravity differences among sauces; other variables such as lipid content or air incorporation may have contributed. These differences may also be partly explained by differences in fermentation time, microbial populations, composition of raw materials, and the value of salt used.

**TABLE 3:** Physical properties of the sturgeon intestine traditional sauce are stored in plastic, pottery, and wooden containers.

Index sauce treatments	Soluble solids (°Brix)		Specific gravity		Salt absorption (%)	
	Zero time	Sixth month	Zero time	Sixth month	Zero time	Sixth month
Pottery	32.38 ± 1.48^aB^	32.39 ± 1.49^aB^	2.65 ± 0.59^aB^	2.68 ± 0.59^aB^	23.24 ± 1.99^aA^	23.24 ± 1.98^aA^
Wooden	32.75 ± 1.58^aB^	32.77 ± 1.57^aB^	2.48 ± 0.48^aB^	2.52 ± 0.57^aB^	23.28 ± 1.82^aA^	23.28 ± 1.85^aA^
Plastic	34.91 ± 1.67^aA^	34.94 ± 1.57^aA^	3.90 ± 0.45^aA^	3.92 ± 0.38^aA^	23.39 ± 1.96^aA^	23.39 ± 1.99^aA^

*Note:* The same letters in the same column show no significant difference (*p* > 0.05). Lowercase letters indicate differences between treatments. Uppercase letters indicate statistical differences for each treatment separately during storage time (Container effect).

Soluble solids ranged from 32.38° to 34.94°Brix. The sauce stored in a plastic container had remarkably greater values of soluble solids (34.94°Brix) (*p* < 0.05) (Table 3). This indicator was lower in wooden treatments (32.27°Brix). The quantity of soluble solids was stable during the keeping time of the sauce. Because this experiment was accomplished at the fermentation termination (After ripening) and on the strained sauce. The apparent stability of the soluble solids over the storage time suggests that protein hydrolysis was completed by the time of fermentation, and these soluble solids compounds are indicative of the extent of proteolysis, which includes the free amino acids and peptides. Proteolysis during fermentation causes the breakdown of large proteins into peptides and amino acids, contributing to both soluble solids and flavor development (Seifzadeh et al. 2025). Nakano et al. (2018) determined soluble solids from 37.85°–50.31°Brix in the salmon sauce generated from flesh, entrails, inedible parts, and smooth roe. This indicator was lower in the available study than in the last study. This discrepancy can be connected to the level of hydrolysis, raw material composition, hydrolysis condition, and filtration.

Salt absorption remained approximately stable (23.24%–23.39%) across pottery, plastic, and wooden containers during storage time (*p* > 0.05) (Table 3). Based on Codex Alimentarius Commission (2018), salt quantity is expected to be 20% in sauce; therefore, salt values (23.24%–23.39%) were admissible in the available study. The salt absorption was stable during the sauce storage. Because salt absorption was carried out at the termination of the fermentation and on the matured and strained sauce, it did not vary during the sauce's keeping. In great salt quantity (6%), the speed of the chymotrypsin hydrolysis response was high. High‐level salt‐created structure alteration is noted as an agent influencing the enzyme reaction dynamics of chymotrypsin. This research used a 25% salt content, which is an important agent in adjusting microbial and enzymatic activities during sauce processing. High salt amounts effectually prevent deterioration and bacterial activity, consequently suppressing corruption and the expansion of unpleasant taste. This generates a particular habitat where only halotolerant or halophilic bacteria can endure actively, allowing the managed failure of proteins and fats required for taste and tissue growth. However, the tall salt degree also impacts the activity of texture intrinsic enzymes. For example, trypsin and pepsin manifest ideal activity at about 5% salt and are considerably silenced at greater concentrations. Conversely, chymotrypsin keeps usability under high‐salt situations, designating a dissimilar answer among proteases. As an outcome, the raised salt levels may restrict the activity of some internal enzymes that participate in proteolysis, diminishing protein disintegration and peptide generation. This is compatible with the smaller resolvable protein quantities detected amid fermentation (Liu et al. 2024). Proteolytic enzymes are crucial to the conversion stages, since they continuously decompose proteins, consisting of linking structures, providing the sauce's taste and odor. While high salt harms the proteolysis rate as it influences enzyme conformation and activity, the slower degradation is good because it makes sure that the proteolysis is controlled, the protein is not completely broken down, and the complex and desirable flavors are created. Also, halotolerant or halophilic proteases, which are adapted to high salt environments, can be more active in such conditions and thus will produce certain breakdown patterns characteristic of the sauce. Lipolytic enzymes are no less important. They convert fats into fatty acids that become even more influential in the flavoring of a sauce that is uniquely distinctive. The same to proteolytic activity, lipolysis is regulated by the salt concentration, which produces enzymes that are adapted to the saline conditions and thus support the necessary enzymatic reactions (Liu et al. 2024). Sakpetch et al. (2022) explained that the salt of the budu specimen extended 23.38%–24.55% overall during the fermentation. Nakano et al. (2018) reported that the salt mass of the salmon sauce generated from flesh, viscera, an inedible section, and flexible roe was 19.33%–23%. In our samples, salt quantity was 23.15%–23.45% at the extremity of the fermentation. Current outcomes were similar to recent studies.

### 3.4 Hydrolysis Rate

The protein hydrolysis degree is applied as an indicator for the rate of fermentation. Table 4 presents hydrolysis rates of 70% in pottery and wooden containers compared with 90% in plastic containers. The considerably higher level in plastic containers shows extra extreme fermentation (*p* < 0.05). Yanohara et al. (2022) proved that the extraction measure was within 60% in the internal organs sauce of white sturgeon. Sakpetch et al. (2022) confirmed that the scale of hydrolysis was attained at 58.39% in budu samples. However, it was 70%–90% in the current research. Perceived disagreement may be related to processing situations and their compounds.

**TABLE 4:** The hydrolysis rate of the sturgeon intestine traditional sauce is stored in plastic, pottery, and wooden containers at the end of the fermentation period.

Treatment index	Pottery	Wooden	Plastic
Hydrolysis rate	70^B^	70^B^	90^A^

*Note:* The same letters in the same row show no significant difference (*p* > 0.05).

### 3.5 Physicochemical Stability

pH is a key agent that influences fermentation. The pH stayed within the harmless scope (5.99 to 6.49) during storage, manifesting constancy regarding sauce treatments. The pH was lowest in the plastic treatment (5.99) and highest in the pottery treatment (6.49) (*p* > 0.05). pH presented considerable differences during the storage period in the refrigerator (*p* < 0.05). However, the pH of samples stored in pottery containers did not show significant changes during storage in the refrigerator (*p* > 0.05). The allowable pH range is 5–6.5 (Codex Alimentarius Commission 2018). Therefore, the pH of experimental samples was acceptable. The pH of fish is neutral. Adding salt causes the pH to decline from 7.0 to 5.11–6.19 at the fermentation termination (Table 5). This is related to the dissociation of amino acids and weightless peptides in the presence of salt. At the beginning of fermentation, the digestive enzyme trypsin performs autolysis, and it remains constant in a neutral environment. Therefore, it would assist in raising protein hydrolysis (Puat et al. 2015). Pottery containers have alkaline properties due to their mineral structure, and storing the product in these containers neutralizes the acidic nature of the sauce (6.49) (Koochakian Sabour 2000). This agent slightly extended pending keeping and achieved 5.99–6.49. It may be related to the enlargement of volatile nitrogenous bases, thiobarbituric acid, and the alkaline attribute of aldehyde (the chief volatile constituent) obtained from the breakdown of the early products of lipid oxidation. Dissimilar aldehydes were generated during prolonged fermentation (Sakpetch et al. 2022). Although acidic products cannot be stored in earthenware containers for a long time because they may cause product spoilage, in the present study, the acidity of the product was not high enough to cause product spoilage within 6 months of storage. Zarei et al. (2012) reported the pH of Mahyaveh in the range of 4.89–7.55. Yanohara et al. (2022) reported a pH of 6.08 in the internal organs sauce of the white sturgeon. pH was determined to be 5.99–6.49 in the trial samples at the end of the keeping period. It had no remarkable dissimilarity from the recent studies. Bacterial fermentation causes acid generation and a pH change. Due to the presence of gastric bacteria and their conformity to these situations, acid production can be expected. Pepsin, protease, and chymotrypsin are internal enzymes. Also, pH is a suitable repressor of their activity. The pH most favorable for chymotrypsin is at 8.0, but hydrolysis can also be performed at pH 7 and 9. The optimum pH for trypsin function is 11.0. pH 7 is the best condition for protease action. However, pH (5.28–5.99) is not suitable for this internal proteolysis enzyme task (Zamani et al. 2023; Siringan et al. 2006). Chymotrypsin and protease can have almost all activity at this pH. In addition, the most favorable temperature for trypsin activity is 50°C, and a significant fall in activity is noted at the lower and upper temperatures. 30°C and 50°C are the best temperatures for protease and chymotrypsin function. Therefore, 30°C–35°C was suitable for protease function, and it can be said that protease has the main role in traditional fermentation.

**TABLE 5:** Physicochemical factors of the sturgeon intestine traditional sauce are stored in plastic, pottery, and wooden containers.

Treatments	Pottery				Wooden				Plastic			
Sampling time (Month)	TVB‐N mg/100 g	PV value meq/kg oil	TBARS mg MDA/kg	pH	TVB‐N mg/100 g	PV value meq/kg oil	TBARS mg MDA/kg	pH	TVB‐N mg/100 g	PV value meq/kg oil	TBARS mg MDA/kg	pH
Zero time	17.24 ± 2.96^gB^	0.79 ± 0.26^dB^	0.77 ± 0.37^cA^	6.19 ± 1.14^aA^	18.26 ± 1.18^gB^	0.96 ± 0.35^dB^	0.97 ± 0.60^gA^	5.26 ± 1.17^cA^	23.34 ± 2.17^gA^	0.89 ± 0.74^cA^	0.99 ± 0.49^eA^	5.11 ± 1.14^cA^
1	33.16 ± 2.75^fB^	2.35 ± 1.15^cB^	2.34 ± 0.92^bA^	6.22 ± 1.40^aA^	33.56 ± 2.87^fB^	2.97 ± 1.26^bB^	2.78 ± 1.12^cdA^	5.39 ± 1.36^cA^	42.46 ± 2.99^fA^	2.75 ± 0.86^bA^	3.71 ± 1.14^abA^	5.20 ± 1.43^cA^
2	53.28 ± 2.93^eB^	3.26 ± 1.54^aB^	3.22 ± 1.15^aA^	6.28 ± 1.81^aA^	52.46 ± 2.29^eB^	3.82 ± 1.81^aB^	3.88 ± 1.41^aA^	5.47 ± 1.28^cA^	64.36 ± 1.84^eA^	4.58 ± 0.74^aA^	4.15 ± 1.38^aA^	5.45 ± 1.21^bcA^
3	60.14 ± 1.50^dB^	3.14 ± 1.13^aB^	3.21 ± 1.23^aA^	6.36 ± 1.70^aA^	59.74 ± 1.78^dB^	3.70 ± 1.43^aB^	3.14 ± 1.21^bcA^	5.72 ± 1.23^bcA^	72.54 ± 1.65^dA^	4.61 ± 0.87^aA^	3.53 ± 1.18^bA^	5.52 ± 1.31^bA^
4	63.16 ± 1.35^cB^	2.89 ± 1.77^abB^	2.88 ± 1.33^aA^	6.42 ± 1.63^aA^	64.82 ± 1.96^cB^	2.58 ± 1.36^bB^	2.58 ± 1.34^deA^	5.79 ± 1.19^bA^	77.16 ± 1.28^cA^	4.75 ± 0.97^aA^	3.24 ± 1.29^bcA^	5.87 ± 1.30^abA^
5	74.16 ± 1.77^bB^	2.55 ± 1.19^bcB^	2.37 ± 1.26^bA^	6.44 ± 1.56^aA^	73.68 ± 1.32^bB^	2.50 ± 1.15^bcB^	2.21 ± 1.64^eA^	5.97 ± 1.21^abA^	84.96 ± 1.77^bA^	4.62 ± 0.99^aA^	3.01 ± 1.43c^cA^	5.95 ± 1.26^aA^
6	89.64 ± 1.10^aB^	2.34 ± 1.28^cB^	1.93 ± 1.35^bA^	6.49 ± 1.24^aA^	90.28 ± 1.25^aB^	2.46 ± 1.16^cB^	1.49 ± 1.55^fA^	6.27 ± 1.15^aA^	94.52 ± 1.63^aA^	4.53 ± 1.17^aA^	2.48 ± 1.40^dA^	5.99 ± 1.19^aA^

*Note:* The same letters in the same column and row show no significant difference (*p* > 0.05). Lowercase letters indicate differences between treatments. Uppercase letters indicate statistical differences for each treatment separately during storage time (Container effect).

Based on the information provided, both PV and TBARS levels for all the samples were within the allowed levels. This suggests that the oxidative stability of the products was preserved within an acceptable range. The TBARS levels, which were indicative of secondary oxidation of lipids, were minimum when the samples were stored in wooden containers (1.49 mg MDA/kg) and maximum when stored in plastic (2.48 mg MDA/kg) (*p* > 0.05). All values for TBARS levels were well below the acceptable limit of 7–8 mg MDA/kg (Seifzadeh 2022). Peroxide values exhibited a similar trend to TBARS levels, with PV value being highest in plastic samples (4.53 meq/kg oil). This indicated an increased level of oxidative rancidity. The index examined was considerably lower in the samples stored in pottery containers (2.34 meq/kg oil) as compared to plastic containers (*p* < 0.05) and wooden containers (2.46 meq/kg oil) (*p* > 0.05) for the sauce. The acceptable limit for PV is 5–10 meq/kg oil (Seifzadeh 2022); therefore, the PV value from the samples was fine. The PV and TBARS values were significantly different during the storage period in the refrigerator (*p* < 0.05) (Table 5). Importantly, the PV and TBARS values were higher in the samples stored in plastic containers, suggesting that plastic storage conditions may be more conducive to oxidative rancidity when compared to wood and ceramics. The proposed link between high salt absorption and lower water activity has significance for lipid oxidation (Seifzadeh and Raoufi 2023). While the lowering of water activity could first inhibit microbial growth, it may also provide a substrate that promotes oxidation. Sakpetch et al. show that the PV value of samples increased from 9.57 meq/kg oil at 0 day to 16.37 meq/kg oil within 60 days of fermentation (*p* < 0.05). Likewise, the initial TBARS value was 3.91 mg MDA/kg, which increased gradually thereafter to 7.26 mg MDA/kg after 60 days of fermentation. Additionally, our results regarding peroxide and tiobarbituric acid levels increasing during the first 2 months in pottery (0.79–3.26 meq/kg oil), plastic (0.89–4.85 meq/kg oil) and wooden (0.96–3.82 meq/kg oil) containers until a later reduction of these levels to 2.34, 4.53, and 2.46 meq/kg oil peroxide, respectively, at the end of the storage period. Thiobarbituric acid followed a similar pattern. It reached 3.22, 4.15, and 3.88 mg MDA/kg after 2 months of storage and 1.93, 2.48, and 1.49 mg MDA/kg after the 6th month of storage. These findings emphasize the relative oxidation of sauce stored in pottery and wooden containers. Our results differ from those of the previous study, and this difference is related to the type of raw material, the sauce storage container, and microbial activities.

TVB‐N is a measure for the development of spoilage (Han et al. 2023). TVB‐N values increased steadily, reaching a peak of 94.52 mg/100 g in plastic containers, at month 6. This value was significantly lower in samples stored in pottery (89.64 mg/100 g) treatments, compared with the wooden (90.28 mg/100 g) and plastic (94.52 mg/100 g) treatments (*p* < 0.05). All values were below the permissible limit of 200 mg/100 g. TVB‐N showed significant differences throughout the storage period in the refrigerator (*p* < 0.05) (Table 5). Developing TVB‐N can be generated by the dissociation of the fish proteins by the inherent proteolytic enzymes and microorganisms. In addition, this factor also depends on the number of bacteria present in the sample (Zang et al. 2020). Total bacterial counts were lower in the sauce stored in wooden and pottery containers than in plastic containers. Therefore, TVB‐N was higher in the sauce stored in the plastic container. The growth of TVB‐N may be due to osmosis, which leads to the substitution of water and soluble nitrogen compounds abandoned from the fish cells (Hjalmarsson et al. 2007). Also, it may be related to the crude matter and the manufacturing situations. Seifzadeh et al. (2025) reported the TVB‐N of traditional sauce stored in glass jars as 13.28–86.17 mg/100 g. TVB‐N was 89.64–94.52 mg/100 g in experimental samples. Our outcomes were higher than the recent study. Zarei et al. (2012) determined the overall mean of TVB‐N in Mahyaveh to be 3098 mg/100 g. Our outcomes were lower than this study. The dissimilarity can be relevant to the ingredients and manufacturing conditions. This suggests that sauce stored in pottery and wooden containers has protein stability.

### 3.6 Color Attributes

Color did not differ significantly over time (*p* > 0.05), indicating visual stability (Table 6). The lightness (L*) values were significantly different among fish sauces stored in pottery (86.45) compared to fish sauces stored in wooden (84.23) and plastic (83.79) containers (*p* < 0.05) at the end of the storage period. No significant difference was observed between wood and plastic (*p* > 0.05). A value showed no significant difference among treatments (*p* > 0.05). However, B value was higher in sauce stored in pottery containers (4.76) compared with the wooden samples (2.99) (*p* < 0.05).

**TABLE 6:** Color properties of sturgeon intestine sauce and traditional sauce stored in plastic, pottery, and wooden containers.

Index	B (red‐green)			A (yellow‐blue)			Lightness (L)		
Treatments sampling time	Pottery container	Wooden container	Plastic container	Pottery container	Wooden container	Plastic container	Pottery container	Wooden container	Plastic container
Zero time	4.96 ± 0.75^aA^	3.25 ± 0.49^aB^	3.76 ± 1.28^aA^	3.37 ± 0.99^aA^	3.19 ± 0.98^aA^	3.13 ± 0.92^aA^	86.72 ± 0.38^aA^	84.45 ± 1.65^aAB^	84.14 ± 1.19^aB^
1	4.95 ± 0.79^aA^	3.22 ± 1.19^aB^	3.71 ± 1.36^aA^	3.36 ± 1.20^aA^	3.17 ± 0.94^aA^	3.13 ± 0.93^aA^	86.70 ± 1.32^aA^	84.39 ± 1.55^aAB^	84.14 ± 1.69^aB^
2	4.90 ± 0.95^aA^	3.22 ± 1.27^aB^	3.71 ± 1.37^aA^	3.32 ± 1.23^aA^	3.11 ± 1.13^aA^	3.13 ± 0.84^aA^	86.66 ± 1.31^aA^	84.35 ± 1.87^aAB^	84.10 ± 1.83^aB^
3	4.88 ± 0.92^aA^	3.17 ± 1.78^aB^	3.68 ± 1.43^aA^	3.30 ± 1.34^aA^	3.03 ± 1.17^aA^	3.10 ± 0.87^aA^	86.65 ± 1.29^aA^	84.37 ± 1.96^aAB^	84.08 ± 1.87^aB^
4	4.82 ± 0.94^aA^	3.16 ± 1.12^aB^	3.61 ± 1.52^aA^	3.24 ± 1.50^aA^	2.91 ± 1.23^aA^	3.03 ± 0.95^aA^	86.58 ± 1.24^aA^	84.30 ± 1.95^aAB^	84.01 ± 2.28^aB^
5	4.80 ± 0.87^aA^	3.07 ± 1.99^aB^	3.58 ± 1.58^aA^	3.16 ± 1.58^aA^	2.83 ± 1.26^aA^	2.97 ± 0.89^aA^	86.50 ± 1.58^aA^	84.25 ± 2.15^aAB^	83.94 ± 2.59^aB^
6	4.76 ± 0.83^aA^	2.99 ± 1.97^aB^	3.55 ± 1.66^aA^	3.14 ± 1.63^aA^	2.76 ± 1.25^aA^	2.95 ± 0.82^aA^	86.45 ± 1.80^aA^	84.23 ± 2.17^aAB^	83.79 ± 2.55^aB^

*Note:* The same letters in the same column and row show no significant difference (*p* > 0.05). Lowercase letters indicate differences between treatments. Uppercase letters indicate statistical differences for each treatment separately during storage time (Container effect).

### 3.7 Sensory Evaluation

**FIGURE 1:**
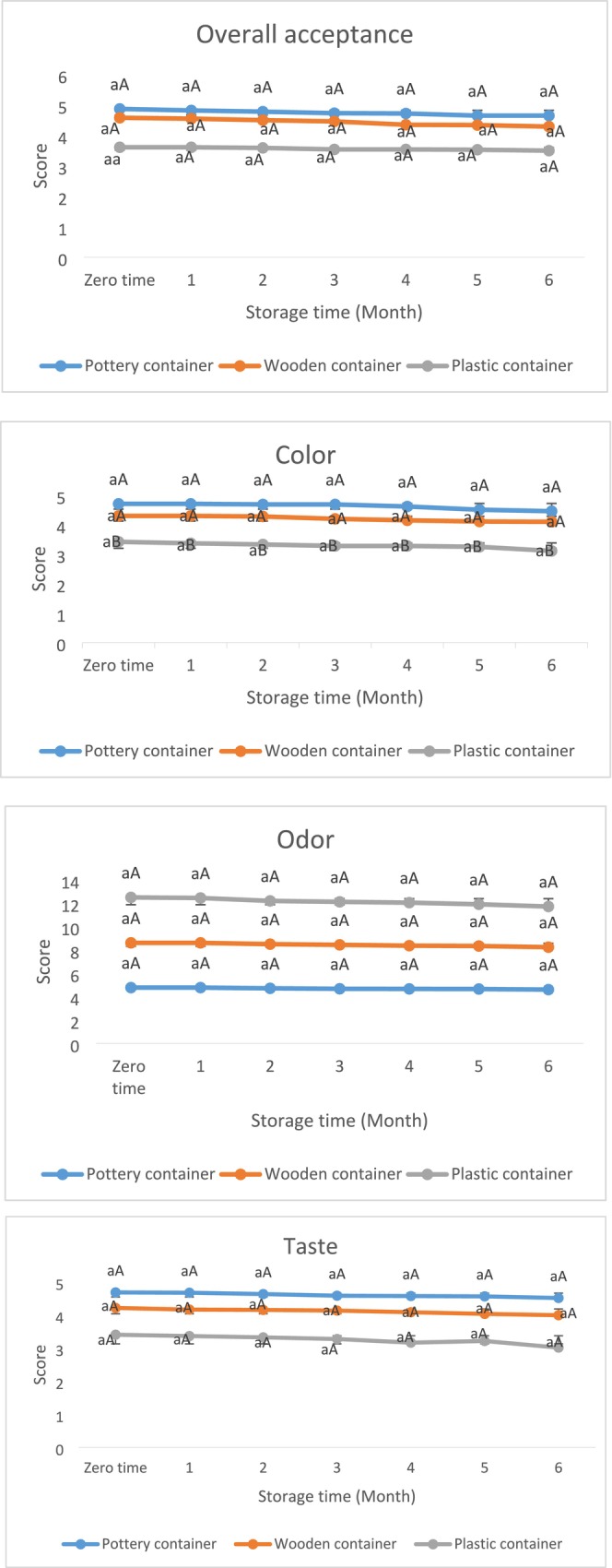
The changes in color, odor, taste, and overall acceptance scores for sturgeon intestine sauce stored in pottery, wooden, and plastic containers over 6 months. The same letters show no significant difference (*p* > 0.05). Lowercase letters indicate differences between treatments. Uppercase letters indicate statistical differences for each treatment separately during storage time (Container effect).

Sensory scores (Figure 1) indicated that fish sauce stored in pottery and wooden containers had higher concentrations consistently through the end of the storage period. All samples appeared clear and visually looked different with color and turbidity in pottery and wooden containers compared with the plastic container. Visual inspections did not show any suspended particles or sediment. Spectrophotometric absorption was negligible, indicating that there was minimal clouding. In addition, microscopic evaluation showed no particulate matter or microbial aggregates. Based on our results, the fish sauce was clear throughout the keeping period, presenting good physical traits and consistency.

As detailed in Table 6 and displayed in Figure 1, sensory features and overall acceptance (Plastic, pottery, and wood at 3.50, 4.65, and 4.29, respectively) were similar across sauce treatments (*p* > 0.05). Overall acceptance decreased slightly over time, but pottery still produced the highest overall acceptance (4.65), while plastic was the lowest (3.50) at month 6 (*p* > 0.05). The samples packed in clay containers (4.52) were rated higher for taste than samples packed in wood (4.00) (*p* > 0.05), and plastic (3.02) (*p* < 0.05). The small increase in taste of the samples could just as easily be attributed to the salt. This improvement is consistent with the various pathways of flavor development through the biochemical breakdown or metabolic pathways of amino acids and fatty acids. During the fermentation process, most likely the flavor compounds came from aromatic and branched‐chain amino acids. These amino acids are precursors to many of the important flavor‐contributing volatiles (aldehydes, alcohols, and esters); therefore, it can be assumed that they contributed to the positive sensory statements of the pottery and wood‐packed samples over the storage time. These compounds tend to be formed by various enzymes, such as branched‐chain amino acid transferase and lipoxygenase (Seifzadeh et al. 2025; Ding et al. 2020). The similarity of sensory conclusions among treatment groups could be explained through consistent biochemical reactions taking place during fermentation, producing positive volatiles. These compounds all originate from protein breakdown, Maillard reactions, and lipid oxidation, which altogether are put together to help make the average aroma and flavor of fish sauce (Sakpetch et al. 2022). The absence of thiobarbituric acid high levels indicates that lipid peroxidation was low to moderate, contributing to the stability of flavor from breakdown during storage. There were also some impacts from the type of container (Seifzadeh et al. 2025). Earthenware containers are not reactive and may have helped stabilize pH and stabilize some of the volatile compounds, allowing aroma retention and some contribution to flavor integrity (Koochakian Sabour 2000). Compared to plastic or wood, clay containers contributed to improved flavor changes. One important point to note is that the clay containers are chemically inert and somewhat porous, which also allows for micro‐ventilation that favorably provides for microbial activity and enzymatic activity, allowing the pottery to stabilize pH and temperature ranges, preventing loss of volatile aroma compounds by absorption or any alteration contributing to the overall flavor characteristics of the sauce. Also, the fermentation temperature (37°C) used in this study was appropriate as it allowed for suitable enzymatic reactions without significantly contributing any less desirable flavors, while still favorably supporting sensory attributes (Ding et al. 2020).

The fact that the scent is very important for buyers and marketing should be taken into consideration. The exploratory products had an enjoyable scent during storage. Samples had no significant differences during the storage period (3.48–4.61). The aroma decreased slightly over time but remained highest in pottery (4.61) and lowest in plastic (3.48) at month 6 (*p* > 0.05). But the sample stored in clay containers had a better smell compared to the other samples, which is due to the smell of clay (4.61). Volatile fatty acids are the principal scent active ingredients of fish sauce, mainly 3‐methylbutanoic, 2‐methylpropanoic, and acetic acids (Sakpetch et al. 2022). Because they are susceptible to oxidation and their subordinate metabolites, in numerous instances, the scent quality diminished that related to oxidation and peroxide development. As regards these indices were within the ordinary limit, the scent alteration of the product did not arise in the available study (Seifzadeh et al. 2025).

The color decreased slightly over time but remained highest in pottery (4.46) and lowest in plastic (3.11) at month 6. The fish sauce of sturgeon gut had red to brown colors, which were caused by the reaction of amino acids and ribose sugar. The substantial volume of the nitrogenous matter in the sauce is unattached amino acids and little peptides, which create a brown color. Since the sugar quantity in fish is small, carbohydrate simulators, like glucose‐6‐phosphate and other substances in the metabolic pathway, can also function as a starter for the Maillard reaction. In addition, the brown color in the sauce was caused by non‐enzymatic browning (Sakpetch et al. 2022). The Maillard Reaction is a complex series of chemical reactions that transpire between amino acids and reducing sugars as a result of proteins undergoing fermentation. The Maillard reaction makes a considerable contribution to the respective color, flavor, and aromatics that develop in food products; fish sauce, for example (Seifzadeh 2022). In fish sauce, the protein‐peptide composition consists largely of fish proteins, which undergo degradation into smaller peptides and free amino acids during fermentation. The peptides are then used as substrates for the Maillard reaction. During fermentation, enzymes and microbes hydrolyze fish proteins into free amino acids. Glycine, lysine, and alanine, for example, are present in fish and produce flavor (Seifzadeh et al. 2025). The carbohydrates in fish tissue can be hydrolyzed and fermented to reducing sugars, which are essential for the Maillard reaction. During the Maillard reaction, the free amino acids react with reducing sugars and form complex browning products that provide a golden to brown color to the fish sauce. This color development provides not only the color but also the sign of flavor development produced during fermentation (Koochakian Sabour 2000). Many factors affect the extent of the Maillard reaction in our fish sauce, including high temperature during fermentation (30°C–35°C) can enhance the process of the Maillard reaction and the color. The pH of fish sauce (5.99–6.49) varies and can enhance reaction rates, as slightly acidic conditions usually enhance the Maillard reaction. Time allowed for fermentation (6‐month fermentation) allows for more extensive interactions of proteins and sugars, developing more robust colors, textures, and flavors (El Hosry et al. 2025).

Based on the considerable protein quantity in the sauce stored in pottery and wooden containers (13.45% and 10.28%), they had a darker and browner color than the sauce stored in plastic containers (9.51%). Protein volume was higher in sauce stored in wooden and pottery containers (13.45%–10.28%). Thus, color was better in these samples (86.45). The color in the sauce specimens was a pleasant state (83.79–86.45). This agent did not cause a considerable decrease in the color of the sauce stored in plastic containers (3.11) compared with the sauce stored in wooden (4.10) and pottery (4.46) jars. The dissimilarity in color in sauce treatments is related to the acid obtained from the function of fermenting bacteria and internal enzyme hydrolysis, and its clarifying trait (Seifzadeh et al. 2025). Moreover, Tonsil soil was applied for sauce purification. It relates to a bleaching clay (often purified bentonite‐based) commonly used to absorb impurities, and for purification in food processing. It may affect sensory properties by adsorbing off‐flavors and pigments, and stabilizing color and flavor. Additionally, it makes the extract clearer (Koochakian Sabour 2000). Wongngam et al. (2025) stated that the aroma of Thai fish sauces produced from anchovies from different sources, including western, eastern, and southern, was 3.55, 2.50, and 2.55, respectively. The odor acquired a great grade in sauce stored in pottery and wooden containers compared with the sauce stored in a plastic container (3.48–4.61). Available outcomes from plastic and wooden containers are in agreement with the recent research. Nakano et al. (2018) explained that the amounts of lightness in a salmon sauce made of muscle, entrails, inedible parts, and soft roe were 32.25–66.79. However, its grades were 83.79–84.45 in the available study (Table 6). Available outcomes were superior to the recent studies. It can be relevant processing conditions, manufacturing containers, bacterial counts, and acid generation during fermentation (Seifzadeh 2022).

## 4 Conclusion

According to this study, the type of storage container plays an important role in the sauce quality (nutritional, microbial, chemical, sensory, and physical) of sturgeon gut sauce during fermentation and storage. Pottery containers performed best, as evidenced by higher protein retention, lower bacterial counts, oxidative stability, and higher sensory characteristics, including taste, aroma, and color, than the other container types. By comparison, plastic containers showed the least nutritional retention with the highest levels of protein hydrolysis, lipid oxidation, and bacterial growth. The application of plastic containers, in this given case, is more concerning for food quality but also possible health concerns due to microplastics leaching, in addition to environmental concerns. The data provide evidence that using pottery containers or similar unlined natural containers could improve quality, safety, and reduce negative environmental impact in a wide variety of fermented seafood products. Ultimately, this study will assist both traditional artisans and modern manufacturers in considering their packaging choices. Further, producers adopting more natural containers could increase overall consumer acceptance, reduce environmental impact, and help preserve cultural heritage related to traditional food fermentation and production practices. Nonetheless, this study has some limitations including the lack of detailed microbial identification, at least beyond total counts, only one salt concentration (25%) and temperature ranges (30°C–35°C), and lack of evaluation of chemical leaching from plastic containers. It was also limited in its sensory assessment as it only used trained panelists and did not involve any consumer sensory analysis. Thus, stronger research should follow using molecular and metagenomic tools to profile microbial communities, replicate fermentation conditions, assess the levels of chemical migration from plastic, include consumers' sensory analysis across broad demographics, and consider novel foods such as containers lined with ceramics or biodegradable options to help improve safety and sustainability in the fermented seafood industry.

## Author Contributions

**Mina Seifzadeh:** Writing (Lead), Original draft (Lead), Methodology (supporting), Supervision (supporting), Investigation (supporting), Experimental design and data curation (supporting). **Anosheh Koochakian Sabour:** investigation (supporting), methodology (supporting), project administration (supporting), supervision (supporting). **Ali Raoufi:** formal analysis (lead), investigation (supporting), resources (supporting), software (supporting), validation (lead), writing – review and editing (supporting).

## Ethics Statement

This study has been ethically approved by and registered in the Agricultural Research Education and Extension Organization (AREEO) of Iran by the registration code 84/685. According to the rules, studies involving sensory analysis do not require additional ethical approval by any other organization or body.

To explain the ethics, a description of how the study was carried out is provided below:

1. The material, method, and ethical conditions of the project were evaluated by the Agricultural Research, Education, and Extension Organization (AREEO), and an approval code was obtained.
2. The present study was not conducted on live fish and was carried out on the by‐products after the processing of the sturgeon.
3. The by‐products were transferred to the processing center under sanitary conditions and in ice‐filled Styrofoam containers.
4. Chemical and microbiological tests were conducted on the by‐products. The safety of the by‐products was confirmed after comparing and matching them with national and international standards.
5. The salt and the other ingredients used in the production of the sauce were of food grade.
6. To conduct the sensory analysis, firstly, microbiological and chemical tests were conducted on the sauce. Secondly, the results were matched to national and international standards. Finally, the sensory analysis was carried out.
7. The risks and the dangers of participation in the sensory analysis were explained to the participants, and informed consent was obtained from every participant.
8. The women participating in the analysis were asked about being pregnant, ruling out the participation of pregnant women in the study.
9. The material, method, and results of the final report were approved by seafood processing and food science reviewers, and then the final approval code was obtained from the Agricultural Research, Education and Extension Organization (AREEO) of Iran.

## Conflicts of Interest

Dr. Kouchakian is retired and is not currently employed by any organization or company (government or private). Dr. Raofi is a student and is not currently employed by any organization or company (government or private). Dr. Seifzadeh is a scientific member of the National Fisheries Research Processing Center (affiliated with the Iranian Fisheries Sciences Research Institute) and has no financial interest in implementing this project. His duty is to conduct research and implement the project. The authors did not receive any money from any government or private institution for implementing this project. This project was not carried out with financial assistance or consulting from the private sector. The ownership of the shares of this project has not been transferred to the private sector for production. This project has not been patented.

## Data Availability

No additional information is available about this article.
